# Micropropagation of *Dipcadi montanum* (Dalz.) Baker (*Asparagaceae*): a rare scapigerous herb

**DOI:** 10.5114/bta.2024.139755

**Published:** 2024-06-25

**Authors:** Sautrik Basu, Emadul Islam, Debraj Chakraborty

**Affiliations:** Post Graduate Department of Botany, Barasat Government College, Barasat, West Bengal, India

**Keywords:** *Dipcadi montanum*, bulbous geophyte, mass propagation, cytology

## Abstract

*Dipcadi montanum* (Dalz.) Baker (*Asparagaceae*) is a rare scapigerous herb endemic to the Western Ghats, a global biodiversity hotspot running parallel to the western coast of India. This study reports the development of a reproducible protocol for mass propagation of this underutilized geophyte using bulb scale and immature leaf base explants. Miniature bulblets were successfully induced from both types of explants after 4 and 8 weeks of culture on full-strength semisolid MS basal medium fortified with 3% sucrose and varying levels of BAP (4.4–17.7 μM) and TDZ (4.5–18.1 μM). The addition of 2.7 μM NAA further enhanced the rate of microbulb induction. Rooting of the 8-week-old bulblets, obtained from both explants, was achieved with more than 90% efficiency on liquid as well as agar-gelled half-strength MS basal medium fortified with varying levels of IBA (2.46–9.84 μM) and NAA (2.68–10.74 μM), with or without 2.32 μM Kinetin. More than 95% of the rooted plants survived the initial acclimatization process under controlled ex-vitro conditions, and a survival rate of over 80% was recorded after 4 weeks of transfer to greenhouse conditions. After a brief dormancy, the regenerants resumed growth in the postmonsoon season and exhibited morphological resemblance to the donor plant. Comparative cytological analysis between the donor and 15 randomly selected regenerants revealed a stable somatic count of 2*n* = 20.

## Introduction

*Dipcadi* Medik. is a genus of bulbous flowering plants in the family Asparagaceae (Subfamily: Scilloideae), comprising approximately 41 species distributed across the Mediterranean region, Madagascar, Africa, and Southwest Asia, with India being one of its centers of diversity (Prabhugaonkar et al., 2009). In India, the genus is represented by ten species, including four varieties, with seven species found in Maharashtra and two in South India (Prabhugaonkar et al., 2009). Many species within the genus *Dipcadi* are considered threatened and are thus prioritized for conservation efforts (Dasgupta and Deb, 1988; Samanta et al., 2023). *Dipcadi montanum* (Dalz.) Baker is a rare plant that grows on the slopes of rocky crevices in scrub jungles of the Western Ghats, Peninsular India, and in a few isolated pockets of the Western Himalayas. Since the plant is not cultivated and is primarily restricted to wild habitats, there is limited information available regarding its ethnobotany. Available literature indicates that *Dipcadi* leaves are used as vegetables (Yadav and Sardesai, 2002), the storage bulbs and immature capsules are edible in Pakistan (Ali and Qaiser, 2005), and the tender leaves are often used as a laxative and as an ointment for wounds in Bahrain (Moussaid et al., 2013). Plants belonging to the family Asparagaceae have been of interest as a potential source of important bioactives, including alkaloids such as bufadienolides and homoisoflavanones, which possess a wide range of medicinal properties (Mottajhipisheh and Stuppner, 2021).

Phytochemical analysis conducted on *Dipcadi krishnadevarayae* and *Dipcadi erythraeum* (native to Arabia and Egypt) revealed the presence of alkaloids, flavonoids, glycosides, and inorganic ions such as sulfates and phosphates (El-Shabrawy et al., 2016; Vijaya Jyothi et al., 2018). Despite its rarity, ethnomedicinal significance, and potential horticultural value, *Dipcadi montanum* has received relatively little attention compared to other species within the genus.

A comprehensive review of the literature indicates a lack of reports on the *in vitro* propagation and regeneration of *D. montanum*. Due to its limited axillary branching and low seed germination percentage, bulbous geophytes with commercial value are traditionally propagated using slow vegetative methods, which can become slower with successive propagation cycles (De Klerk, 2012). However, the application of *in vitro* propagation methods can significantly accelerate the process and maintain a continuous year-round supply of true-to-type planting material. Therefore, considering these factors, the present investigation was undertaken to develop a rapid protocol for the mass propagation of this underutilized bulbous geophyte.

## Materials and methods

### Plant material

Ten *D. montanum* plants in the reproductive phase were collected from the outskirts of Satara city (District: Satara; State: Maharashtra; India; elevation: ±1000 m above sea level). The bulbs were carefully washed with running tap water and planted in the experimental garden of Barasat Government College, Barasat (District: North 24 Parganas; State: West Bengal; India; elevation: ± 11 m above sea level), using a mixture of garden soil and organic manure (obtained from a commercial nursery) in a 2 : 1 proportion. The plant was identified using standard taxonomic literature and by direct comparison with herbarium specimens available at the Central National Herbarium (CAL) (Botanical Survey Of India, AJCB Indian Botanic Garden, Howrah-3, West Bengal). A voucher specimen was deposited in the Departmental Herbarium of Barasat Government College (District: North 24 Parganas; State: West Bengal; India).

### Surface disinfection of bulb scales and fruits

Outer scales (2.5–3 cm in length) were carefully removed from five randomly selected healthy *D. montanum* bulbs. The scales were washed thoroughly under running tap water and then washed with a 1% (v/v) solution of Tween-20 for 10 min to remove visible dirt. To reduce fungal contaminants, the explants were treated with a 1% (w/v) solution of Carbendazim for 25 min, followed by decontamination with aqueous HgCl_2_ (0.1%; w/v) for 12 min in front of a laminar flow hood. After a thorough wash in sterile double-distilled water (three times), the explants were placed on appropriate media. For the aseptic germination of seeds, mature fruits were collected from healthy plants. The fruits underwent similar treatment as described above for the bulb scales.

### Culture establishment

For aseptic germination, the surface-disinfected fruits were longitudinally split into two halves, and the black discoid seeds were carefully dissected out and placed on the germination medium. For the induction of bulblets, sterilized bulb scales (abaxial surface touching the medium) and later leaf base explants (0.5–1.0 cm in length) obtained from *in vitro* germinated seedlings were inoculated on the appropriate medium.

### Media composition and culture conditions

For the aseptic germination of *Dipcadi* seeds, halfstrength Murashige and Skoog’s basal medium (MS) (Murashige and Skoog, 1962) containing 3% sucrose and 0.8% agar (bacteriological grade) was used. Cultures were maintained in the dark (at 22°C) to simulate the soil conditions. For induction of bulblets from bulb scale and leaf base explants (obtained from 15 days old germinated seedlings) semisolid MS supplemented with varying levels of 6-Benzyl aminopurine (BAP) (4.4–17.7 μM) and Thidiazuron (TDZ) (4.5–18.1 μM) in combination with/without 2.7 μM α-Naphthalene acetic acid (NAA) was used.

For root induction in the *in vitro* grown bulblets obtained from both explants, half-strength MS medium (both agar-gelled and liquid) supplemented with 3% sucrose, 0.75% agar (in the case of semisolid MS), and varying concentrations of Indole-3-butyric acid (IBA) (2.46–9.84 μM) and α-Naphthalene acetic acid (NAA) (2.68–10.74 μM) were used in combination with or without 2.32 μM Kinetin.

In all cases, the pH values of the media were adjusted to 5.7 with 1N HCl/1N NaOH after the addition of all growth regulators. This was followed by autoclaving at 121°C for 15 min. All cultures were maintained at 22±2°C under a 16/8 photoperiod provided by cool white fluorescent lamps (Photosynthetic photon flux density (PPFD) of 60 μmol m^-2^s^-1^). For all experiments, subculturing was performed after 4 weeks, and all observations were recorded after intervals of 4 or 8 weeks. All photographs were taken using a Zeiss Stemi 508 stereo zoom microscope. All chemicals and reagents used were of analytical grade and were obtained from Himedia (Mumbai, India), E. Merck (Germany), Sigma-Aldrich (USA), and Qualigens Fine Chemicals (Mumbai, India).

### Acclimatization and ex vitro transfer

To acclimatize the *in vitro* regenerants, plantlets with well-developed roots and 3–4 healthy leaves were washed thoroughly to remove traces of agar and planted in polythene-covered plastic pots containing a mixture of sterile soil and sand (1 : 1). The plants were maintained at 25°C under a 12/12 photoperiod and 65% relative humidity (RH). After four weeks of hardening, the surviving plants were transferred to earthen pots containing garden soil in a greenhouse, where they were maintained at 30°C and 60% RH.

### Cytological analysis of the regenerants

For cytological analysis, root tips (approximately 0.5 cm long) from the donor plant and 15 randomly selected *in vitro* regenerants (approximately 10 months old) were collected and pretreated with saturated paradichlorobenzene (PDB) with a trace of Esculine at 14°C for 5 h. Pretreated root tips were fixed overnight in freshly prepared chilled aceto-ethanol (1 : 3; v/v) at 12–14°C. Fixed root tips were hydrolyzed in 1 NHCl at 37°C for 10 min and eventually stained in 2% propionicorcein for 1 hour. The images were captured using a Carl Zeiss trinocular microscope fitted with a CCD camera (Zeiss, Axiocam). Measurements were made from 5 well-scattered metaphase plates using the Zeiss Axiovison L.E.4 software package. Chromosome measurements were carried out following previously published methods (Basu and Jha, 2011). Chromosomes were classified following the system proposed by Levan et al. (1964). The categorization of karyotype asymmetry was performed according to the classification system proposed by Stebbins (1971).

### Statistical analysis

All experiments were repeated three times. Each experimental set was in triplicate, and each replicate consisted of identical culture vessels (25 mm × 150 mm culture tubes made of borosilicate glass) containing the same quantity of media (25 ml) and the identical number of explants (3 explants/vessel). The data were subjected to one-way analysis of variance (ANOVA) using SPSS version 22.0 for Windows (Chicago, USA), and means were separated through Duncan’s multiple range test at 5% probability level (Duncan, 1955).

## Results and discussion

**Fig. 1:**
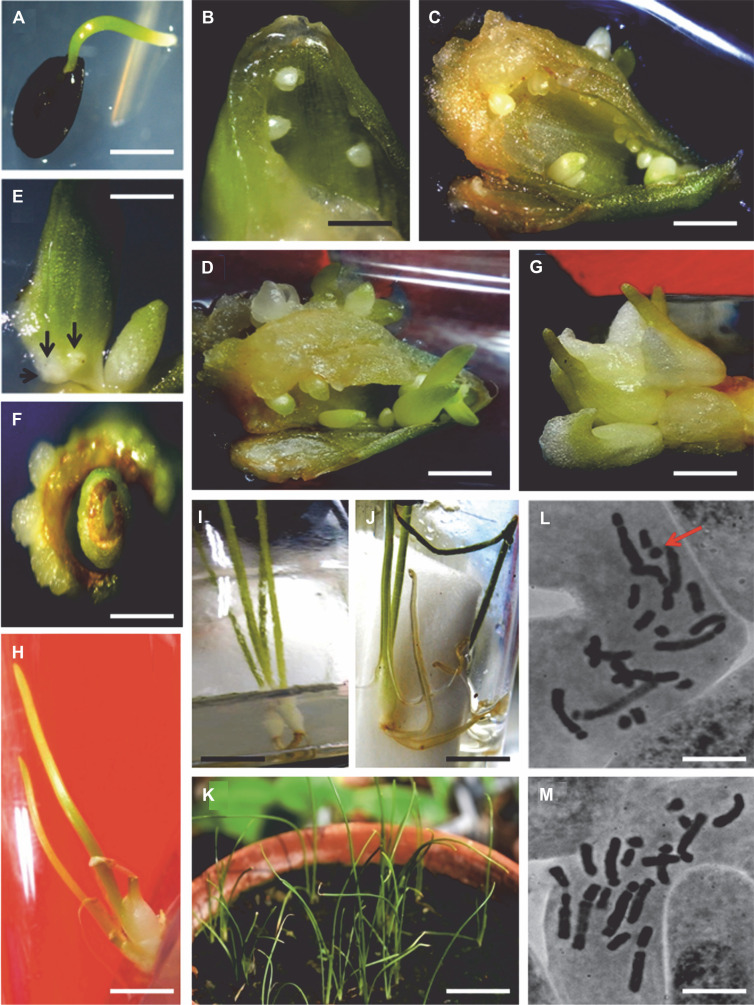
Micropropagation of *Dipcadi montanum* from bulbs scale and leaf base explants; A) *in vitro* germination of *Dipcadi montanum* seeds (5 days old) in half-strength MS basal medium fortified with 3% sucrose and 0.8% agar (scale bar: 1 cm); B) induction of microbulbs from bulb scale explants after 2 weeks of culture (scale bar: 1 cm); C) adventitious bulblet formation from a single bulb scale after 8 weeks of culture on nutrient medium (MS, 3% sucrose, 6.8 μM BAP + 2.7 μM NAA) (scale bar: 1 cm); D) formation of adventitious bulblet clusters from a single bulb scale after 8 weeks of culture on nutrient medium (MS, 3% sucrose, 9 μM TDZ + 2.7 μM NAA) (scale bar: 1 cm); E) swelling and appearance of small protuberances from the edges of immature leaf base explants (after 10 days of incubation) (scale bar: 1 cm); F) formation of small bulblet primordia from the basal edges of leaf base explants (after 2 weeks of culture) (scale bar: 1 cm); G) cluster of bulblets with leaves appearing on top (scale bar: 1 cm); H) a single 8-week-old bulblet with well-developed leaves ready for rooting (scale bar: 1 cm); I) rooting of microbulbs on semisolid half-strength MS, fortified with 3% sucrose, 9.80 μM IBA (scale bar: 1 cm); J) *in vitro* rooting of microbulbs on liquid half-strength MS, fortified with 3% sucrose, 9.80 μM IBA, and 2.32 μM Kinetin (scale bar: 1 cm); K) regenerated *Dipcadi montanum* plants growing under natural light in the greenhouse (after 6 weeks) (scale bar: 2 cm); L) mitotic metaphase from root tip meristematic cells of the donor plant showing 2*n* = 20 chromosomes along with a solitary B chromosome (indicated by arrow) (scale bar: 10 μm); M) mitotic metaphase from root tip meristematic cells of randomly selected regenerants showing a nonsymmetric karyotype consisting of 2*n* = 20 chromosomes without the presence of any B’s (scale bar: 10 μm)

Establishing contamination-free cultures has always been a challenging task for bulbous geophytes, primarily due to the presence of surface-localized contaminants that are difficult to eliminate. Results obtained in the present investigation also indicate that the bulb scale explants possessed internal contaminants, leading to nearly 30% of the inoculated bulb scales being contaminated. On the other hand, the surface-sterilized seeds of *D. montanum* were 95% free of contaminants and germinated (in the dark) within 7 days of incubation on half-strength MS basal medium supplemented with 3% sucrose and 0.8% agar (Fig. 1A). Contamination is a serious and enduring problem in tissue culture work. However, it can be mitigated by using explants from *in vitro*grown sources, such as immature leaf bases. Several nonbulb explants have been previously used for the *in vitro* propagation of bulbous geophytes (Gayathri and Gopal, 2007; Arzu and Basdogan, 2015). Results obtained during the present investigation reveal that, in addition to bulb scales, *in vitro*-grown leaf bases can be successfully utilized as a starting material for *in vitro* culture.

Within 7 days of inoculation, the colorless bulb scales became green, swelled considerably, and exhibited initial signs of morphogenesis. Dome-like structures appeared on the abaxial surface of the green bulb scales, particularly near the wound site (Fig. 1B). The orientation of the explants with their abaxial sides in contact with the medium is superior to other orientations (Askari et al., 2018). Formation of bulblet primordia was also noted near the basal edges of the immature leaf bases after 3 weeks of inoculation (Fig. 1E, Fig. 1F) in all treatments supplemented with growth regulators (Table 1). Hormone-free basal media did not evoke any positive response in either type of explant (Table 1). When used individually, TDZ at varying levels of 4.5–18.1 μM was more efficient compared to various concentrations of BAP (4.4–17.7 μM) – Table 1.

**Table 1:** Effect of BAP (6-benzylaminopurine), TDZ (thidiazuron) and 2.7 μM NAA (α-naphthaleneacetic acid) on bulblet induction of *Dipcadi montanum* using Bulb scale and leaf base explants

Growth regulators used			Bulb scale			Leaf base		
BAP [μM]	NAA [μM]	TDZ [μM]	response [%]	number of bulblets produced (after 4 weeks)	number of bulblets produced (after 8 weeks)	response [%]	number of bulblets produced (after 4 weeks)	number of bulblets produced (after 8 weeks)
00	00	00	NR	00	00	NR	00	00
4.4	–	–	39.8	3.6 ± 1.07 ^j^	8.3 ± 0.76 ^j^	33.6	2.7 ± 0.05 ^l^	4.8 ± 0.32 ^k^
6.8	–	–	74.7	4.9 ± 0.92 ^h^	9.8 ± 1.09 ^g^	70.9	3.9 ± 0.10 ^j^	7.2 ± 0.68 ^i^
8.8	–	–	83.5	6.2 ± 1.41 ^g^	11.8 ± 1.69 ^e^	80.4	6.7 ± 0.54 ^e^	7.5 ± 0.82 ^c^
13.3	–	–	79.6	6.2 ± 0.36 ^a^	10.9 ± 1.29 ^de^	74.6	5.8 ± 1.05 ^e^	9.1 ± 0.08 ^c^
17.7	–	–	74.8	5.1 ± 0.06 ^bc^	9.8 ± 1.18 ^f^	70.3	4.9 ± 0.16 ^b^	8.7 ± 0.71 ^c^
–	–	4.5	86.7	6.0 ± 1.64 ^f^	8.7 ± 1.02 ^i^	78.3	4.8 ± 0.46 ^i^	8.3 ± 0.54 ^g^
–	–	6.8	89.4	6.2 ± 1.26 ^e^	9.4 ± 2.01 ^h^	81.8	5.3 ± 0.55 ^h^	8.9 ± 0.60 ^e^
–	–	9.0	91.8	4.8 ± 0.46 ^h^	7.6 ± 1.12 ^k^	88.9	3.1 ± 0.14 ^k^	5.1 ± 0.22 ^j^
–	–	13.6	90.2	5.9 ± 0.55 ^g^	12.3 ± 1.34 ^f^	89.3	4.8 ± 0.36 ^i^	9.5 ± 0.59 ^h^
–	–	18.1	94.3	7.1 ± 0.68 ^d^	14.6 ± 2.07 ^c^	84.7	7.9 ± 0.93 ^c^	10.7 ± 0.93 ^b^
4.4	2.7	–	51.8	5.6 ± 0.55 ^g^	10.8 ± 1.34 ^f^	48.3	4.8 ± 0.36 ^i^	7.5 ± 0.59 ^h^
6.8	2.7	–	69.4	5.9 ± 0.68 ^d^	11.6 ± 2.07 ^c^	64.8	5.1 ± 0.93 ^c^	9.7 ± 0.93 ^b^
8.8	2.7	–	73.8	7.7 ± 1.12 ^a^	9.8 ± 1.19 ^b^	71.9	6.8 ± 0.75 ^b^	8.3 ± 1.02 ^a^
13.3	2.7	–	61.6	6.9 ± 0.29 ^c^	9.3 ± 1.09 ^a^	54.6	5.7 ± 0.37 ^ab^	8.0 ± 1.03 ^a^
17.7	2.7	–	57.8	6.3 ± 1.84 ^b^	8.6 ± 1.97 ^a^	48.9	4.6 ± 1.05 ^a^	7.4 ± 1.22
–	2.7	4.5	83.7	4.4 ± 0.62 ^i^	6.8 ± 0.92 ^n^	71.6	4.1 ± 0.48 ^f^	6.6 ± 0.56 ^f^
–	2.7	6.8	84.6	5.9 ± 0.45 ^gh^	7.4 ± 0.82 ^l^	82.4	5.5 ± 0.62 ^g^	7.1 ± 0.44 ^h^
–	2.7	9.0	98.7	8.2 ± 1.12 ^a^	16.5 ± 1.19 ^b^	91.2	8.7 ± 0.75 ^b^	10.3 ± 1.02 ^a^
–	2.7	13.6	89.3	4.3 ± 1.32 ^c^	7.6 ± 1.10 ^d^	84.2	3.9 ± 0.69 ^c^	6.9 ± 1.91 ^d^
–	2.7	18.1	81.2	3.2 ± 0.99 ^b^	5.3 ± 1.62 ^a^	78.8	2.9 ± 0.89 ^a^	4.8 ± 0.06 ^a^

Results are mean ± SE of 3 replicates; means followed by same letters in each column are not significantly different at 5% probability level using Duncan’s multiple range test (DMRT); NR – no response

Both bulb scale and leaf base explants exhibited better responsiveness and produced more bulblets when exposed to varying levels of TDZ (4.5–18.1 μM) compared to varying levels of BAP (4.4–17.7 μM). At low concentrations of BAP (8.8 μM), a maximum of 4.9 and 9.8 bulblets were recorded after 4 and 8 weeks of culture, respectively, from a single bulb scale. Immature leaf base explants, on the other hand, yielded 3.9 and 7.2 bulblets after 4 and 8 weeks of culture, respectively. BAP at a concentration of 8.8 μM yielded comparatively better results for both types (Table 1).

Above 8.8 μM, however, the number of bulblets declined considerably in both types of explants. Higher concentrations of BAP were not as effective and yielded fewer bulblets. Even at low concentrations, TDZ evoked a better response for both types of explants. TDZ at a concentration of 4.5 μM resulted in the formation of 6.0 and 8.7 bulblets from a single scale after 4 and 8 weeks of culture, respectively. For leaf base explants, 4.8 and 8.3 bulblets were obtained after 4 and 8 weeks, respectively (Table 1). There was a steady increment in the number of bulblets with progressively higher doses of TDZ used (Table 1).

Although BAP has been used for adventitious bulblet formation and mass propagation of several bulbous geophytes due to its low cost, stability, and high efficacy (Arzu and Basdogan, 2015; Muraseva and Novikova, 2018), the unquestionable superiority and effectiveness of TDZ over other cytokinins have been reported (Pai and Desai, 2018). TDZ, a substituted phenyl urea compound, has been reported to mimic both auxin and cytokinin effects on the growth and differentiation of cultured explants. This non-purine cytokinin-like compound has been shown to promote shoot/bulblet initiation in several bulbous geophytes (Sahoo et al., 2018; Taha et al., 2018). Although auxins are known to inhibit the accumulation of cytokinins, the addition of auxins in the culture medium has also been found to promote the growth and regeneration of bulbous geophytes (Arzu and Basdogan, 2015; Deswiniyanti and Lestari, 2020; Marković et al., 2023).

In the present investigation, the incorporation of NAA at a low concentration (2.7 μM) along with varying levels of individual cytokinins (BAP and TDZ) resulted in an improvement in the number of bulblets obtained from both types of explants (Table 1; Fig. 1C, Fig. 1D). BAP at a concentration of 4.4 μM when combined with 2.7 μM NAA, resulted in a higher number of bulblets from both types of explants after 4 and 8 weeks of culture, respectively (Table 1). A combination of 9.0 μM TDZ in combination with 2.7 μM NAA yielded the highest number of bulblets (16.5 and 10.3) from a single bulb scale and a solitary shoot base, respectively, after 8 weeks of culture. The percentage of explant response was also found to be maximum (98.7% in the case of scales and 91.2% in the case of shoot bases) in the aforementioned combination of TDZ and NAA (Table 1). This is in agreement with the results obtained previously in hybrid Lilium (var. Red Alert) (Taha et al., 2018).

During the present investigation, it was observed that after 8 weeks of culture, all the regenerated bulblets developed leaves. About 50% of the regenerated bulblets also exhibited the formation of closed scales (attached to the basal plate) with leaves arising on the top (Fig. 1G). Similar observations have been recorded earlier in hyacinth lily *Hyacinthus orientalis* var. pink pearl (Pierik and Ruibing, 1973). Although the formation of secondary bulblets was not noticed during the present study, *in vitro* grown bulblets obtained can be effectively cryopreserved, encapsulated in alginate beads, or used as alternative propagules for direct ex vitro transfer. It has been reported earlier that *in vitro* grown miniature bulbs have great advantages as propagules since they can be readily removed from the culture vessel, stored *ex vitro* for long periods without precaution, and successfully planted in soil just like normal bulbs (George et al., 2007).

In the course of the present investigation, bulblet induction occurred at a constant sucrose concentration of 3%. After 8 weeks of culture, the average diameter of the bulblets obtained from both types of explants ranged from 0.2 to 1.3 cm, and the average fresh weight of the microbulbs ranged from 0.03 to 0.75 g. A high concentration of sucrose in the medium is highly favorable for bulblet formation and growth in several bulbous geophytes like *Lachenalia viridifolia* (Maślanka and Kapczyńska, 2022) and *Frtillaria meleagris* (Marković et al., 2021). It has been reported previously that a high concentration of sucrose (5% or more) increases sink activity and enhances cell enlargement by maintaining osmotic potential in the cell, which eventually increases the size of bulblets in *Allium cepa* and *Lilium orientalis* (Kahane et al., 1992; Youssef et al., 2019). However, in the present investigation, bulblet induction occurred without any anomaly at a constant sucrose concentration of 3%, which is in concurrence with the results obtained previously in *Lilium mackliniae* (Sahoo et al., 2018).

Eight-week-old *in vitro* grown bulbs (average diameter: 1.1 cm) with 2–3 well-developed leaves (possessing normal stomata) were subjected to rooting (Fig. 1H). Hormone-free media were not conducive for root induction. The number of roots developed as well as their length depended on the growth medium and the type of auxin used. Liquid half-strength MS basal medium was found to be more conducive for *in vitro* rooting and induced healthy roots in more than 90% of bulblets. This may be due to the fact that liquid medium has a greater stimulatory effect on growth due to better uptake of nutrients and phytohormones by growing plants (Mehrotra et al., 2009). Furthermore, liquid media eliminates the usage of agar, which, in addition to its high cost, often imparts deleterious effects on the growth of cultured tissues (Lucyszyn et al., 2007).

Out of the two auxins employed for *in vitro* rooting, IBA was found to be more efficient in comparison to NAA. Half-strength liquid MS fortified with 9.84 μM IBA induced 3.7 roots having a length of 3.4 cm after 4 weeks of culture (Fig. 1I). The addition of Kinetin at a concentration of 2.32 μM along with varying levels of IBA and NAA enhanced the rooting efficiency of the bulblets (Table 2). The addition of 9.84 μM IBA and 2.32 μM Kinetin to a half-strength liquid MS medium resulted in the production of the maximum number of roots (4.8) after 4 weeks of culture. On the other hand, the combination of 2.68 μM NAA and 2.32 μM Kinetin was less efficient in comparison to the IBA-Kinetin combination and produced 4.1 short and stout roots after 4 weeks of culture (Table 2; Fig. 1J). The supplementation of the medium with higher concentrations of NAA (> 5.37 μM) resulted in a gradual decrease in rooting efficiency and encouraged callus formation from the basal ends of the unrooted bulblets.

**Table 2:** Effect of IBA (indole-3-butyric acid) and NAA (α-naphthaleneacetic acid) with/without 2.32 μM kinetin on *in vitro* rooting of *Dipcadi montanum* bulblets (after 4 weeks)

Growth regulators used			1/2 MS (semi solid)		1/2 MS (liquid)	
IBA [μM]	kinetin [μM]	NAA [μM]	mean number of roots produced/bulblet (after 4 weeks)	mean root length [cm]	mean number of roots produced/bulblet (after 4 weeks)	mean root length [cm]
00	00	00	NR	–	NR	–
2.46	00	00	2.1 ± 0.23 ^g^	2.9 ± 0.22 ^h^	2.5 ± 0.15 ^h^	3.4 ± 0.45 ^g^
4.92	00	00	2.4 ± 0.27 ^f^	3.1 ± 0.28 ^g^	3.1 ± 0.24 ^g^	3.9 ± 0.32 ^e^
9.84	00	00	2.9 ± 0.16 ^d^	3.6 ± 0.37 ^f^	3.7 ± 0.36 ^e^	3.4 ± 0.23 ^f^
2.46	2.32	00	3.1 ± 0.30 ^c^	3.9 ± 0.44 ^cd^	3.9 ± 0.23 ^de^	4.7 ± 0.38 ^c^
4.92	2.32	00	3.8 ± 0.28 ^b^	3.8 ± 0.47 ^e^	4.5 ± 0.48 ^ab^	4.9 ± 0.48 ^b^
9.84	2.32	00	4.2 ± 0.36 ^a^	2.8 ± 0.27 ^h^	4.8 ± 0.42 ^a^	5.3 ± 0.27 ^a^
00	00	2.68	2.6 ± 0.17 ^e^	4.5 ± 0.45 ^a^	2.7 ± 0.33 ^h^	4.4 ± 0.31 ^d^
00	00	5.37	3.1 ± 0.29 ^c^	3.9 ± 0.23 ^d^	3.6 ± 0.31 ^f^	4.6 ± 0.17 ^c^
00	00	10.74	2.9 ± 0.14 ^d^	3.7 ± 0.31 ^f^	3.4 ± 0.40 ^cd^	4.9 ± 0.27 ^b^
00	2.32	2.68	3.9 ± 0.39 ^bc^	4.0 ± 0.19 ^bc^	4.1 ± 0.22 ^e^	3.9 ± 0.20 ^f^
00	2.32	5.37	3.2 ± 0.22 ^b^	4.1 ± 0.38 ^b^	3.8 ± 0.38 ^cd^	3.6 ± 0.16 ^b^
00	2.32	10.74	2.3 ± 0.31 ^e^	3.9 ± 0.25 ^cd^	2.7 ± 0.29 ^bc^	3.2 ± 0.39 ^c^

Results are mean ± SE of 3 replicates; means followed by same letters in each column are not significantly different at 5% probability level using Duncan’s multiple range test (DMRT); NR – no response

Regenerated plantlets from both explant sources with well-developed roots were subjected to hardening. After about 4 weeks of acclimatization under laboratory conditions, a survival rate of more than 95% was recorded. After about 6 weeks, the surviving regenerants were transferred to the greenhouse and exposed to natural light. The tissue-cultured plants were transferred to *ex vitro* conditions in winter months (December–January), and a survival rate of more than 80% was recorded. After about 8 weeks of *ex vitro* transfer, the regenerants exhibited slow growth, and about 48% of plants became leafless and went into dormancy. Normal growth, however, resumed in the postmonsoon period when the emergence of new leaves took place, and all regenerants exhibited appreciable growth and resembled the donor plants (Fig. 1K).

In the case of bulbous geophytes, dormancy is an adaptive response that enables their survival during harsh winter months (Gillespie and Volaire, 2017). Among other environmental factors, temperature has been reported to play a predominant role in controlling the growth, development, and flowering of bulbous geophytes (Khodorova and Boitel-Conti, 2013). It has been reported earlier that a “warm-cold-warm” sequence is required for the completion of the life cycles of bulbous geophytes, especially temperate geophytes. The loss of leaves in tissue-cultured plants during winter may be a normal adaptive event required for the completion of their life cycle (Alipour et al., 2021).

Cytological analysis conducted on root tip meristems of the donor plant and 15 randomly selected regenerants revealed a constant diploid number of 2*n* = 20 (Fig. 1L, Fig. 1M). The karyotype characters of both the donor and the tissue-cultured regenerants were found to be similar. The total chromatin length (TCL) was measured to be 108.4 ± 0.06 μm in the mother plant and 106.8 ± 1.31 μm in the regenerated plants, with a longest/smallest ratio (L/S) of 5.1. The lengths of the longest and smallest chromosomes were 19.9 ± 0.07 μm and 3.90 ± 0.16 μm in the donor plant and comparable results were obtained in the regenerants, with the longest and smallest chromosomes measuring 19.4 ± 1.20 μm and 3.80 ± 0.91 μm, respectively. The total form percent (TF%) in both cases was recorded to be 31.80. Both the donor plant and the regenerants exhibited a highly asymmetrical karyotype (3c category) (Stebbins, 1971), consisting of 1 pair of metacentric chromosomes, 8 pairs of subterminal chromosomes, and 1 pair of very small telocentric chromosomes. It is noteworthy that the donor plant exhibited one B chromosome (Karyotype formula: 2m + 16St + 2t + 1B), which disappeared in the regenerants, resulting in a karyotype formula slightly different from that of the donor (2m + 16St + 2t + 0B) (Fig. 1L). Root tip cells from all 15 regenerated plants exhibited the presence of 20 A chromosomes only (Fig. 1M).

The genus *Dipcadi* has an enigmatic status among other members of the Asparagaceae family due to occurrences of multiple basic numbers, variable somatic counts (both at the diploid and polyploid levels), dysploidy, polysomaty, and a variable number of B chromosomes (Naik, 1974). *D. montanum* has been reported to exhibit various counts (2*n* = 10, 12, and 20), and the origin of B chromosomes in *Dipcadi* has been variously interpreted (Naik, 1974). B chromosomes are known for their instability, and cases of B chromosome elimination in tissue-cultured regenerants have been reported earlier in *Cymbopogon martinii* (Sreenath and Jagadishchandra, 1988).

The rapid protocol for mass production and regeneration of clonally uniform plant material described in this study is crucial for the future breeding and commercial utilization of bulbous geophytes. These plants are valuable sources of a wide range of biologically active compounds, making them important in ethnomedicine. However, overexploitation and destructive harvesting practices have negatively impacted natural populations. The regeneration protocol presented here can help in the mass propagation and conservation of these geophytes, including *D. montanum*, and can be applied to genetic transformation and other technologies.

## Conclusion

The protocol allows for stable, year-round production of true-to-type *D. montanum* plants. Our findings demonstrate that immature leaf bases, in addition to bulb scale explants, can be used for *in vitro* propagation. The microbulblets obtained can be encapsulated, stored for long periods, and used as alternative propagules. The regenerated plants can be used for large-scale cultivation as ornamentals or for obtaining pharmaceutical products. *D. montanum*, being rare and highly habitat-specific, has considerable horticultural and medicinal value. Like other members of the genus, the bulbs and leaves of *D. montanum* could be important sources of bioactive compounds. Therefore, chemical screening of this underutilized plant is recommended.
